# *In Vitro* Reconstitution of Functional Type III Protein Export and Insights into Flagellar Assembly

**DOI:** 10.1128/mBio.00988-18

**Published:** 2018-06-26

**Authors:** Hiroyuki Terashima, Akihiro Kawamoto, Chinatsu Tatsumi, Keiichi Namba, Tohru Minamino, Katsumi Imada

**Affiliations:** a Graduate School of Science, Osaka University, Toyonaka, Osaka, Japan; b Graduate School of Frontier Biosciences, Osaka University, Suita, Osaka, Japan; c RIKEN Quantitative Biology Center, Suita, Osaka, Japan; Harvard University

**Keywords:** type III secretion system, bacterial flagellum, *in vitro* reconstitution, inverted membrane vesicle, protein transport

## Abstract

The type III secretion system (T3SS) forms the functional core of injectisomes, protein transporters that allow bacteria to deliver virulence factors into their hosts for infection, and flagella, which are critical for many pathogens to reach the site of infection. In spite of intensive genetic and biochemical studies, the T3SS protein export mechanism remains unclear due to the difficulty of accurate measurement of protein export *in vivo*. Here, we developed an *in vitro* flagellar T3S protein transport assay system using an inverted cytoplasmic membrane vesicle (IMV) for accurate and controlled measurements of flagellar protein export. We show that the flagellar T3SS in the IMV fully retains export activity. The flagellar hook was constructed inside the lumen of the IMV by adding purified component proteins externally to the IMV solution. We reproduced the hook length control and export specificity switch in the IMV consistent with that seen in the native cell. Previous *in vivo* analyses showed that flagellar protein export is driven by proton motive force (PMF) and facilitated by ATP hydrolysis by FliI, a T3SS-specific ATPase. Our *in vitro* assay recapitulated these previous *in vivo* observations but furthermore clearly demonstrated that even ATP hydrolysis by FliI alone can drive flagellar protein export. Moreover, this assay showed that addition of the FliH_2_/FliI complex to the assay solution at a concentration similar to that in the cell dramatically enhanced protein export, confirming that the FliH_2_/FliI complex in the cytoplasm is important for effective protein transport.

## IMPORTANCE

The type III secretion system (T3SS) is the functional core of the injectisome, a bacterial protein transporter used to deliver virulence proteins into host cells, and bacterial flagella, critical for many pathogens. The molecular mechanism of protein transport is still unclear due to difficulties in accurate measurements of protein transport under well-controlled conditions *in vivo*. We succeeded in developing an *in vitro* transport assay system of the flagellar T3SS using inverted membrane vesicles (IMVs). Flagellar hook formation was reproduced in the IMV, suggesting that the export apparatus in the IMV retains a protein transport activity similar to that in the cell. Using this system, we revealed that ATP hydrolysis by the T3SS ATPase can drive protein export without PMF.

## INTRODUCTION

Type III secretion systems (T3SSs) are critical to the function of bacterial injectisomes and flagella. The injectisome is a bacterial protein transporter used to deliver virulence proteins into host cells for infection. The flagellum is a motile organelle critical for infection by many pathogens and is constructed by the T3SS built into the base of the flagellum.

**FIG 1:**
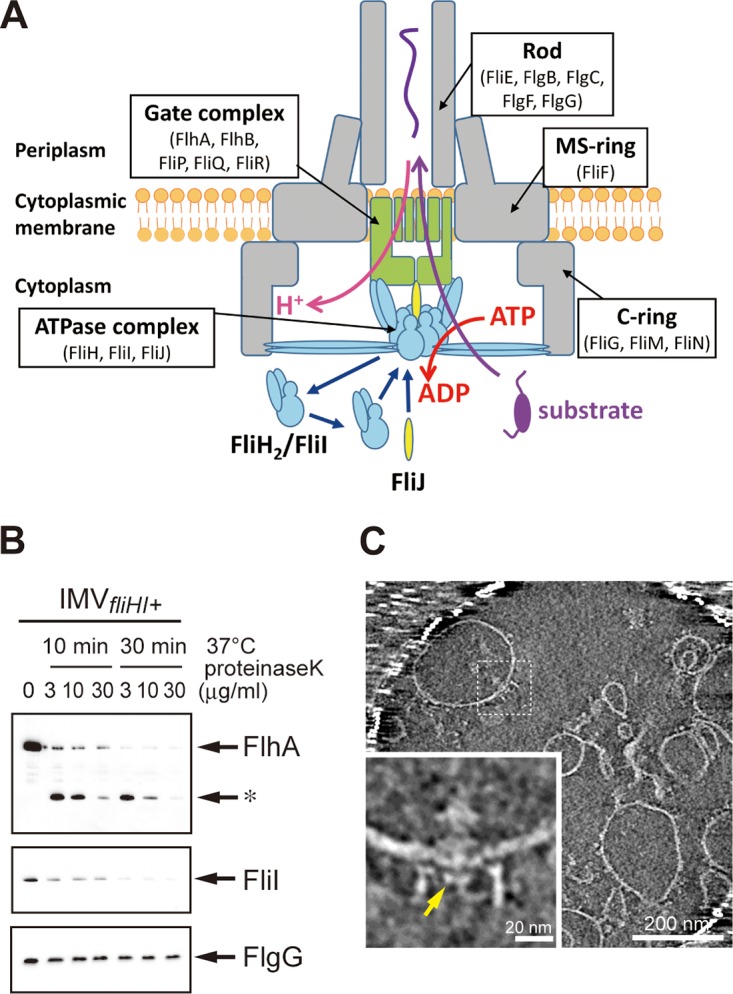
Verification of inverted membrane vesicle (IMV) formation. (A) Schematic drawing of the flagellar protein export apparatus in the basal body. The transmembrane gate components are colored in green. FliH and FliI are shown in cyan, and FliJ is shown in yellow. Substrate proteins are shown in purple. The FliI_6_/FliJ complex is attached to the gate complex and the C-ring through FliH. (B) Digestion of flagellar proteins in the IMV with proteinase K. The IMV solution was mixed with proteinase K (3, 10, or 30 µg/ml) and incubated at 37°C. The reactants were sampled after 0, 10, and 30 min of incubation and were then analyzed by immunoblotting. FlhA (top panel) and FliI (middle panel) were degraded by proteinase K, but FlgG (bottom panel) was retained, indicating that the membrane is surely inverted. The asterisk indicates a partially degraded intermediate of FlhA. (C) Electron cryotomographic image of the IMVs. The lower left panel is a magnified image of the basal body in the IMV. The disk-like density is indicated by a yellow arrow.

The bacterial flagellum consists of a rotary motor spanning the cell envelope and a filamentous axial structure extending out from the cell surface. The axial structure is a tubular, helical protein assembly composed of more than 20,000 protein subunits of about 10 different proteins. These axial proteins and their assembly scaffold proteins, such as FlgJ, FlgD, and FliD, are translocated via the flagellar T3SS, the flagellar protein export apparatus, across the cytoplasmic membrane into the central channel of the growing flagellum. The flagellar protein export apparatus consists of the transmembrane gate complex formed by FlhA, FlhB, FliP, FliQ, and FliR and the cytoplasmic ATPase complex composed of FliH, FliI, and FliJ (1–3) (Fig. 1A). It was thought that FliO is also one of the transmembrane gate proteins, but a recent study revealed that it only facilitates the assembly of the export gate complex (4). Except for FliO, these proteins share sequence homologies with those of injectisome T3SSs in pathogenic bacteria (5). The gate complex is located in the central pore of the basal body MS-ring, and the ATPase complex is bound just below the gate complex in the cytoplasm through the interactions with the gate and the basal body C-ring (6). FliI is a Walker-type ATPase (7) and is homologous to the α and β subunits of F_1_-ATPase in structure (8). FliI and FliJ together form the FliI_6_/FliJ complex that is similar to the F_1_-α_3_β_3_γ complex, suggesting that the FliI_6_/FliJ complex hydrolyzes ATP in a way similar to F_1_-ATPase (9). FliH forms a homodimer similar to the peripheral stalk of V-ATPase (10) and binds to the N-terminal domain of FliI in a way similar to F- and V-type ATPases (11, 12). While FliH is indispensable for efficient formation of the FliI_6_/FliJ complex at the flagellar base (13), FliH also regulates the ATPase activity of FliI negatively by forming the FliH_2_/FliI complex and suppresses the hexamerization of FliI in solution (11, 12). The FliH_2_/FliI complex binds to late export substrates in complex with their cognate chaperones (14–16) and ensures the interaction between the chaperone-substrate complex and FlhA (17), but the details are unclear.

Flagellar protein transport is primarily driven by proton motive force (PMF) (18, 19). FlhA has an ion channel activity, and the FlhA-FliJ interaction enables effective utilization of PMF for protein export (20). Since infrequent ATP hydrolysis by FliI ATPase with the E211D substitution is sufficient for processive protein export for flagellar formation, the energy of ATP hydrolysis by FliI is thought to be required primarily for gate activation (18, 19, 21, 22). A study on the virulence T3SS ATPase InvC has suggested that the energy of ATP hydrolysis is used to unfold substrate proteins for export (23). However, it has been shown that the ATPase activity of InvC is not essential for protein unfolding (24). Therefore, it is still controversial how these two types of energy are used in the protein export mechanism.

Despite many genetic and biochemical studies on the T3SSs, the molecular mechanism of protein transport is still obscure due to difficulties in accurate measurements of protein transport under precise control of measurement conditions *in vivo*. Therefore, an *in vitro* transport assay system with easy control of measurement conditions is needed for further in-depth mechanistic understanding of protein transport. The inverted membrane vesicle (IMV)-based assay has been used for studying protein translocation across the cell membrane (25), such as the Sec machinery (26) and the twin arginine translocation (TAT) machinery (27), but has never been applied to supramolecular complexes such as the T3SS. We have developed an IMV-based flagellar protein transport assay system that enables accurate measurements of protein transport under well-controlled conditions. The export apparatus in the IMV preserves the protein transport activity. The formation of the flagellar hook about 55 nm in length was reproduced in the IMV, and the export apparatus in IMV retains the protein transport function at a similar level as that in the cell. Surprisingly, even ATP hydrolysis by FliI alone was able to drive flagellar protein export without PMF. We discuss the molecular mechanism of protein transport on the basis of this novel protein transport assay.

## RESULTS

### Preparation of inverted membrane vesicles.

The IMVs were prepared from a *Salmonella* Δ*fliT* Δ*flgD* Δ*flhB* mutant strain expressing FlhB(N269A) and FlhD/FlhC. We used a *fliT* null and FlhD/FlhC-overexpressed mutant because deletion of the *fliT* gene and overexpression of FlhD/FlhC increase the number of the flagellar basal bodies per cell (28). We also introduced a *flgD* null mutation allele because we used purified FlgD to evaluate the protein transport activity of IMV. FlgD has most frequently been used for our *in vivo* export analysis of the flagellar T3SS because of the sensitivity and quality of our polyclonal anti-FlgD antibody for measuring the amount of FlgD. FlgD is a scaffolding protein required for the flagellar hook formation. FlgD forms a cap complex at the distal end of the growing hook, and the hook protein FlgE is incorporated into the growing hook just beneath the cap complex (29, 30). Therefore, the hook is not formed without FlgD. FlgD and FlgE are rod-hook-type proteins and need no specific chaperones for export. The flagellar export apparatus switches export specificity from rod-hook-type proteins to filament-type proteins upon completion of hook assembly (2); hence, we introduced the FlhB(N269A) mutation to lock the export apparatus in the rod-hook-type protein export mode (31). FlhB undergoes autocleavage between N269 and P270, and the autocleavage is a critical event for the export specificity switch. Therefore, the FlhB(N269A) mutation completely inhibits the cleavage and locks the export apparatus in the rod-hook-type protein export mode.

The cells were converted to spheroplasts by treatment with EDTA and lysozyme to remove the outer membrane and the peptidoglycan layer, respectively, and were then disrupted by a high-pressure homogenizer to produce inside-out vesicles. The vesicles were purified by sucrose density gradient centrifugation. The vesicles contained FlhA and FlhB (membrane components); FliH, FliI, and FliJ (soluble components); FliF (MS-ring component); and FliG, FliM, and FliN (C-ring components) as judged by immunoblotting (see Fig. S1A in the supplemental material). To verify the membrane orientation, the vesicles were incubated with proteinase K, followed by immunoblotting with polyclonal anti-FlhA, anti-FliI, and anti-FlgG antibodies. FlhA and FliI were degraded (Fig. 1B, top and middle panels), indicating that they are present outside the membrane vesicles. In contrast, FlgG (a rod protein) was resistant to proteolysis (Fig. 1B, bottom panel), indicating that it is inside the vesicles. FlhA and FliI are located on the cytoplasmic side of the export apparatus, whereas FlgG is located in the periplasm. Thus, we concluded that the orientation of the membrane vesicles is inside-out. The formation of the IMV was further confirmed by electron cryotomography (ECT). We observed the flagellar basal body embedded in the membrane with the C-ring exposed on the outside of the vesicle (Fig. 1C). A disk-like density identified as the cytoplasmic domain of FlhA in the previous ECT analysis of *Salmonella* and Campylobacter jejuni (6, 32) was observed within the C-ring (Fig. 1C), suggesting that the basal body in the IMV retains the membrane components of the export apparatus. No clear spherical density corresponding to the ATPase complex (33) was observed below the disk-like density. However, we often found some density probably corresponding to a partially disrupted ATPase complex during IMV preparation.

10.1128/mBio.00988-18.1 FIG S1  Verification of the IMV formation. Immunoblotting analysis showing that the flagellar protein export apparatus component proteins are retained in the IMV. The normal IMV (IMV_*fliHI+*_) contained FlhA and FlhB (the export gate components); FliF, FliG, FliM, and FliN (the basal body components); and FliH, FliI, and FliJ (the soluble components). The FliHI-deficient IMV (IMV_Δ*fliHI*_) contained those other than FliH or FliI. Download FIG S1, TIF file, 1.6 MB. Copyright © 2018 Terashima et al. 2018 Terashima et al. This content is distributed under the terms of the Creative Commons Attribution 4.0 International license.

### IMV preserves the protein transport activity.

**FIG 2:**
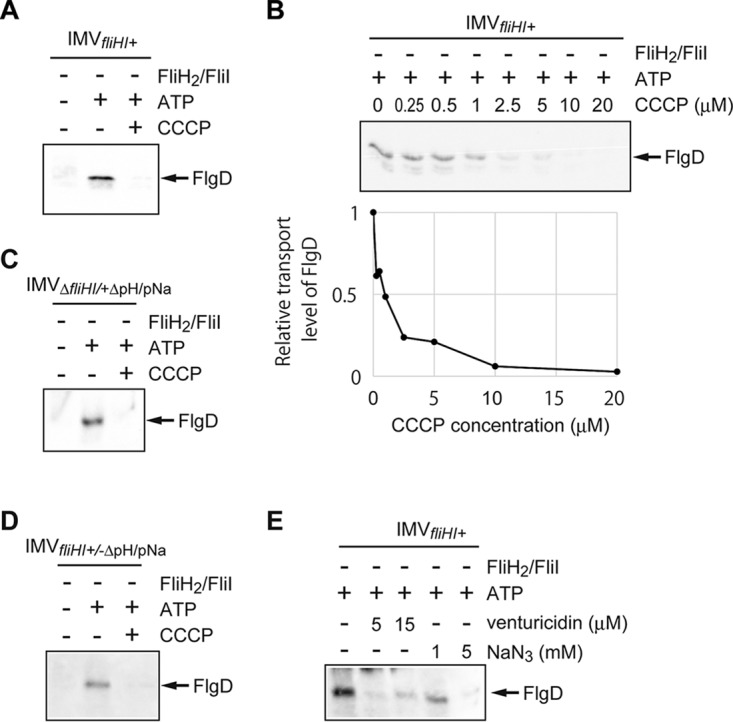
Evidence for inverted membrane vesicles (IMVs) preserving the flagellar protein export activity. *In vitro* protein transport assays were conducted with IMV_*fliHI+*_ (normal IMVs; *fliHI*^+^*/*+ΔpH/pNa) (A, B, and E), IMV_Δ*fliHI/*+ΔpH/pNa_ (Δ*fliHI/*+ΔpH/pNa) (C), or IMV_*fliHI*+/−ΔpH/pNa_ (*fliHI*^*+*^*/−*ΔpH/pNa) (D). FlgD transported into the IMV was analyzed by immunoblotting with polyclonal anti-FlgD antibody. (A) The FlgD transport required ATP and was inhibited by CCCP. The transport assay was conducted with (+) or without (−) 5 mM ATP or 10 µM CCCP. The concentrations of FlgD and FliJ in the reaction mixture were 4 µM and 0.25 µM, respectively. (B) The FlgD transport was suppressed with the increase of CCCP concentration (upper panel). The transport levels of FlgD relative to that without CCCP are shown (lower panel). The concentrations of FlgD, FliJ, and ATP in the reaction mixture were 4 µM, 0.25 µM, and 5 mM, respectively. (C to E) Effect of PMF generated by endogenous F_o_F_1_-ATP synthase on the protein transport into the IMV. (C and D) FlgD transport assay for IMV_Δ*fliHI/*+ΔpH/pNa_ (C) and IMV_*fliHI*+/−ΔpH/pNa_ (D). The transport assay was conducted with (+) or without (−) ATP (5 mM) and CCCP (10 µM). The concentrations of FlgD and FliJ in the reaction mixture were 4 µM and 0.25 µM, respectively. The FlgD transport required ATP and was inhibited by addition of CCCP. (E) The FlgD transport was suppressed by the F_o_F_1_-ATP synthase inhibitors. The transport assay was carried out with venturicidin (5 or 15 µM) or NaN_3_ (1 or 5 mM). The leftmost lane is a control. The concentrations of FlgD, FliJ, and ATP in the reaction mixture were 4 µM, 0.25 µM, and 5 mM, respectively.

The protein transport activity of the export apparatus in the IMV was examined by measuring the amount of FlgD transported into the vesicles. After incubation of the IMVs with purified FlgD in a buffer solution containing 20 mM Tris-HCl, pH 7.5, and 125 mM KCl, followed by proteinase K treatment to digest the external proteins, the transported proteins were monitored by immunoblotting with polyclonal anti-FlgD antibody. FliJ was added in the reaction mixture in all experiments except for the experiments below, because it is essential to efficiently couple PMF with protein export (21). FlgD was transported into the IMVs in the presence of ATP, Mg^2+^, and FliJ in the external solution (Fig. 2A). No FlgD transport was observed for the IMVs prepared from the *flhB* null mutant cells (IMV_Δ*flhB*_), which do not have protein export activity *in vivo* (18) (Fig. S2A). In agreement with previous *in vivo* experiments (18, 19), protein transport was suppressed by a proton-specific ionophore, carbonyl cyanide *m*-chlorophenylhydrazone (CCCP) (Fig. 2A and B). These results indicate that the export apparatus in the IMVs preserves the export activity.

10.1128/mBio.00988-18.2 FIG S2  FlgD was transported depending on Mg-ATP. (A) *In vitro* protein transport assay of FlhB-deficient IMVs (IMV_Δ*flhB*_). No flagellar protein was transported into the IMVs derived from the *flhB* null mutant. The transport assay was conducted with (+) or without (−) 5 mM ATP or 10 µM CCCP. The concentrations of FlgD, the FliH_2_/FliI complex, and FliJ in the reaction mixture were 4 µM, 1.5 µM, and 0.25 µM, respectively. (B) ATP and Mg^2+^ are essential for the protein transport. The transport assay was performed with MgCl_2_ (5 mM), ATP-MgCl_2_ (5 mM), ADP-MgCl_2_ (5 mM), AMPPNP-MgCl_2_ (5 mM), ATP (5 mM), or ATP-CaCl_2_ (5 mM). The concentrations of FlgD and FliJ in the reaction mixture were 4 µM and 0.25 µM, respectively. (C) Transport assay under various concentrations of ATP (upper panel). The transport levels of FlgD relative to that with 5 mM ATP are shown (lower panel). The FlgD transport increases with the increase of ATP concentration. The concentrations of FlgD, FliJ, and MgCl_2_ in the reaction mixture were 4 µM, 0.25 µM, and 5 mM, respectively. Download FIG S2, TIF file, 2.4 MB. Copyright © 2018 Terashima et al. 2018 Terashima et al. This content is distributed under the terms of the Creative Commons Attribution 4.0 International license.

PMF across the cytoplasmic membrane is maintained by proton pumping by the reverse reaction of endogenous F_o_F_1_-ATP synthase in our assay system, although the initial PMF was produced by using IMVs filled with a solution containing 20 mM morpholinoethanesulfonic acid (MES)-NaOH, pH 6.0, and 300 mM NaCl. Mg^2+^ and ATP were both indispensable for FlgD transport in the IMV assay, and Mg^2+^ could not be replaced by Ca^2+^ (Fig. S2B). No FlgD was transported into the IMV when ADP or AMPPNP was used instead of ATP (Fig. S2B), and the amount of transported FlgD depended on the ATP concentration (Fig. S2C). This Mg^2+^-ATP dependence was also detected for the IMVs prepared from the *fliH-fliI* null mutant cells (IMV_Δ*fliHI/+*ΔpH/pNa_) (Fig. 2C) or in the IMVs filled with the same solution as the external buffer (IMV_*fliHI*+/−ΔpH/pNa_) to deplete the initial pH and ion concentration gradients (Fig. 2D). Moreover, the FlgD transport was suppressed by the addition of CCCP for IMV_Δ*fliHI/+*ΔpH/pNa_ and IMV_*fliHI*+/−ΔpH/pNa_ (Fig. 2C and D). These results suggest that endogenous ATPases, such as F_o_F_1_-ATP synthase, generate PMF using the energy of ATP hydrolysis. Addition of F_o_F_1_-ATP synthase inhibitors strongly suppressed FlgD transport, supporting the contribution of F_o_F_1_-ATP synthase to the PMF generation (Fig. 2E). To confirm the contribution of F_o_F_1_-ATP synthase, we altered the F_o_F_1_-ATP synthase locus (subunits α, β, γ, δ, ε, A, B, C, and I) of *Salmonella* strain STH002 (Δ*flhB* Δ*flgD* Δ*fliT* Δ*fliHI*) to tetracycline resistance genes (*tetRA*) and prepared the IMV (IMV_ΔFoF1-Δ*fliHI*_). Deletion of F_o_F_1_-ATP synthase considerably decreased FlgD transport (see Fig. 5A), suggesting that the protein transport by the IMV is mainly driven by PMF generated by the reverse reaction of F_o_F_1_-ATP synthase.

### The FliH_2_/FliI complex in solution is essential for efficient protein transport.

**FIG 3:**
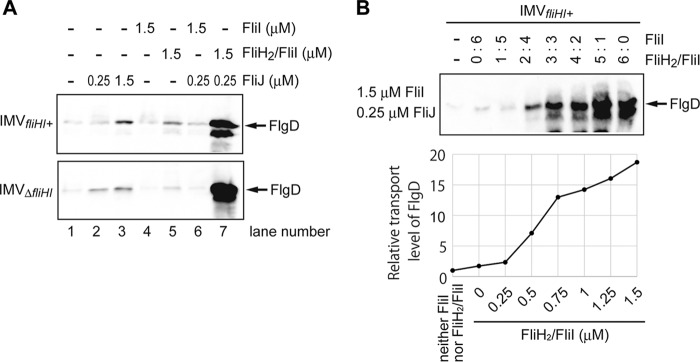
Additive effect of the soluble components of the flagellar protein export apparatus on FlgD transport. FlgD transported into the IMV was detected by immunoblotting. (A) Transport assay with or without soluble component proteins using the normal IMV (*fliHI*^+^) (upper panel) or the FliHI-deficient IMV (Δ*fliHI*) (lower panel). The concentrations of FlgD and ATP in the reaction mixture were 4 µM and 5 mM, respectively. The FlgD transport was markedly increased by addition of both FliJ and the FliH_2_/FliI complex. Individual addition of FliJ or the FliH_2_/FliI complex also facilitated the FlgD secretion to some extent. (B) The transport assay in the presence of FliI and the FliH_2_/FliI complex at various ratios (upper panel). The transport levels of FlgD relative to that without FliI or the FliH_2_/FliI complex are shown (lower panel). The FlgD transport was enhanced by the FliH_2_/FliI complex but not by FliI. The total concentration of FliI was kept at 1.5 µM. The FliJ concentration was adjusted to 1/6 of the FliI concentration. The concentration of ATP in the reaction mixture was 5 mM. The leftmost lane is the negative control (FliI and the FliH_2_/FliI complex were not added).

While the FliI_6_ ring complex associates with the basal body through the interactions of FliH with FlhA and FliN, FliI shows turnovers between the basal body and the cytoplasmic pool in an ATP-independent manner (34), raising the possibility that the FliH_2_/FliI complex present in the cytoplasm is needed for the protein export. To examine this hypothesis, we added the FliH_2_/FliI complex with FliJ to the external solution and found that the FlgD transport into the IMVs was dramatically enhanced (Fig. 3A, upper panel). The transport enhancement was also observed for the IMVs prepared from the *fliH-fliI* null mutant cells (IMV_Δ*fliHI*_) (Fig. 3A, lower panel). These results show that the FliH_2_/FliI complex externally added in solution can assemble onto the export gate complex to form a functional export apparatus. Individual addition of either FliJ or the FliH_2_/FliI complex promoted FlgD transport only slightly (Fig. 3A), which is consistent with previous observations that *fliH-fliI* double null mutant and *fliJ* null mutant strains both show a weakly motile phenotype (18, 19). In contrast, addition of FliI alone did not affect FlgD transport at all (Fig. 3A). These findings indicate that the FliH_2_/FliI complex and FliJ cooperatively act on the protein export mechanism. It is possible that FliH/FliI could fall off during IMV preparation because the FliI density is lost in cryotomograms of lysed cells in the previous studies (33). Then, we preincubated IMVs with the FliH_2_/FliI complex and conducted the transport assay. Preincubation alone showed only a slight enhancement of the FlgD transport (Fig. S3). The effect of the FliH_2_/FliI complex in solution containing purified FliJ was further examined by changing the concentration of the FliH_2_/FliI complex. The transport assay was performed in solution containing FliI and the FliH_2_/FliI complex at various molar ratios with the total amount of FliI kept constant. The concentration of FliJ was adjusted to 1/6 of the total FliI concentration because a small amount of FliJ facilitates the ATPase complex formation but an excess amount of FliJ inhibits FliI hexamer formation (9). The level of FlgD transport was increased considerably with an increase in the amount of the FliH_2_/FliI complex (Fig. 3B). Therefore, we conclude that the FliH_2_/FliI complex in solution is essential for efficient protein export.

10.1128/mBio.00988-18.3 FIG S3  Effect of preincubation with the FliH_2_/FliI complex on the FlgD transport. The transport assay was conducted with (+) or without (−) the FliH_2_/FliI complex (1.5 µM) using the IMVs preincubated with (+) or without (−) the FliH_2_/FliI complex (1.5 µM) and ATP (5 mM). Preincubation of the IMV with the FliH_2_/FliI complex and/or ATP was carried out at 37°C for 30 min. Then, the preincubated IMVs were collected by ultracentrifugation (100,000 × *g*, 30 min) and used for the transport assay. The concentrations of FlgD, FliJ, and ATP in the reaction mixture were 4 µM, 0.25 µM, and 5 mM, respectively. The FlgD transport was dramatically increased by addition of both FliJ and the FliH_2_/FliI complex regardless of preincubation with the FliH_2_/FliI complex and/or ATP. The preincubation itself showed only a small increase in the amount of transported FlgD. Download FIG S3, TIF file, 1.1 MB. Copyright © 2018 Terashima et al. 2018 Terashima et al. This content is distributed under the terms of the Creative Commons Attribution 4.0 International license.

### FlgD facilitates FlgE export.

**FIG 4:**
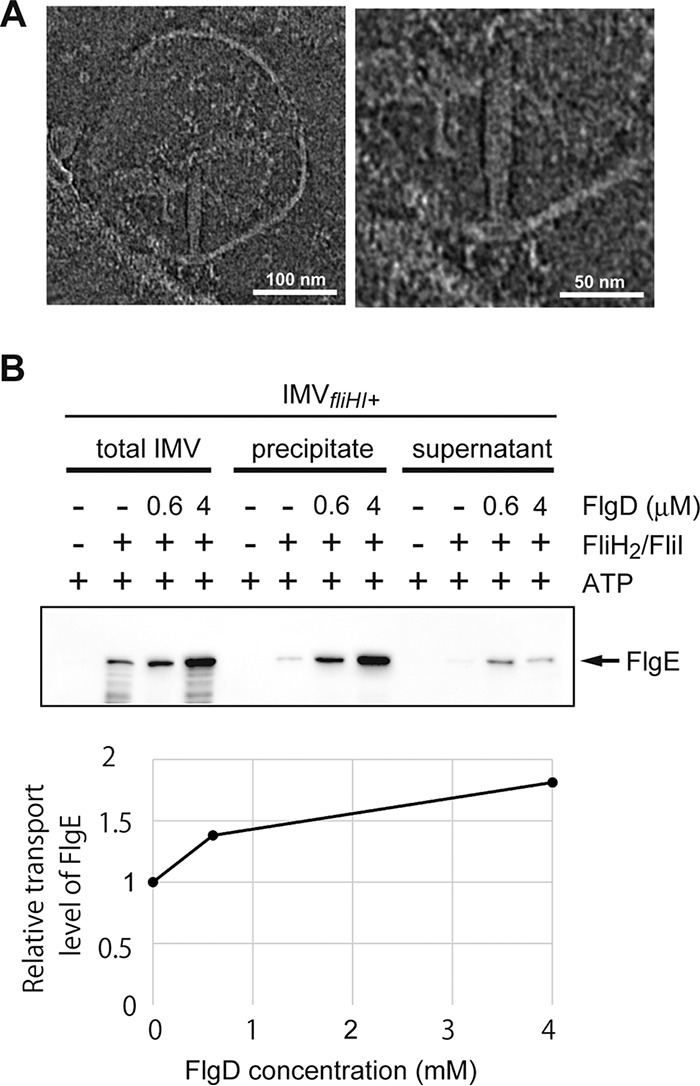
The hook formation inside the IMV. (A) Electron cryotomographic images of the hook formed on the basal body inside the IMV. A representative image is shown in the left panel, and its magnified image is shown in the right panel. The transport assay was conducted with FlgD (4 µM), FlgE (4 µM), the FliH_2_/FliI complex (1.5 µM), and FliJ (0.25 µM) using the normal IMV (*fliHI*^+^). The concentration of ATP in the reaction mixture was 5 mM. (B) FlgE transport is facilitated by FlgD (upper panel). The transport levels of FlgE relative to that in the absence of FlgD are shown (lower panel). The transport assay was conducted with (+) or without (−) FlgD (0.6 µM or 4 µM) and the FliH_2_/FliI complex (1.5 µM). The concentrations of FlgE, FliJ, and ATP in the reaction mixture were 4 µM, 0.25 µM, and 5 mM, respectively. The concentration of K^+^ (KCl and ATP/2K-KOH) in the reaction mixture was adjusted to 133 mM. FlgE transported into the IMV was analyzed by immunoblotting. To analyze the hook formation, we collected the basal body. Part of the reactant was centrifuged (150,000 × *g* for 30 min) after treatment with proteinase K and Triton X-100. The supernatant and the precipitate were analyzed by immunoblotting. In the presence of FlgD at a sufficiently high concentration, most of the FlgE transported into the IMV was detected in the precipitate, indicating that the transported FlgE formed the hook structure.

We next examined the transport of another substrate, FlgE, to investigate hook formation inside the vesicle. We added purified FlgE with or without FlgD in the external solution and performed the *in vitro* protein transport assay. In the presence of FlgD, the transported FlgE monomers assembled into the hook structure on the basal body, resembling those seen in unperturbed cells (Fig. 4A and Fig. S4), indicating that hundreds of FlgE molecules were processively transported into the IMV via a single export apparatus to form a bona fide hook. Interestingly, the amount of transported FlgE was increased by about two times in the presence of FlgD, indicating that FlgD facilitates FlgE export into the IMVs in addition to chaperoning assembly (Fig. 4B).

10.1128/mBio.00988-18.4 FIG S4  Negative-staining electron micrographs of the basal bodies purified from the IMVs after transport assay, without substrate proteins (A), with FlgE (B), and with FlgD and FlgE (C). Images in panels A and B show only rods but no hooks, but long hooks are formed in panel C. The concentrations of FlgD, FlgE, the FliH_2_/FliI complex, FliJ, and ATP in the reaction mixture were 4 µM, 4 µM, 1.5 µM, 0.25 µM, and 5 mM, respectively. Download FIG S4, TIF file, 2 MB. Copyright © 2018 Terashima et al. 2018 Terashima et al. This content is distributed under the terms of the Creative Commons Attribution 4.0 International license.

### *In vitro* hook length control.

The length of the wild-type hook is regulated at about 55 (±6) nm by FliK, a molecular ruler that measures the hook length. After reaching its mature length, FliK interacts with FlhB to switch the export specificity from rod-hook-type to filament-type proteins (2). We tried to reproduce the hook length control and the export specificity switch in the IMV prepared from a *Salmonella* Δ*fliT* Δ*flgD* Δ*flhB* mutant strain expressing wild-type FlhB and FlhD/FlhC by adding purified FliK at various concentrations into the reaction mixture. After a 1-h reaction, we purified the hook-basal bodies from the IMV and measured the hook length by negative-staining electron microscopy (EM). Polyhooks with various lengths were produced without FliK, but with the increase of FliK concentration, the average length became shorter and the length distribution became narrower (Fig. S5). In the presence of 4 µM FliK, the average length was successfully controlled at about 55 (±22) nm, suggesting that no soluble component other than FliK is necessary for the hook length control and substrate specificity switch. The standard deviation (SD) of the hook length was larger than that of the wild-type hook but was similar to that for the cells overproducing FlgE (35). The FlgE concentration used for this assay (4 µM) corresponds to about 4,000 molecules/cell, and this is presumably much higher than the cellular level. The large SD of the hook length may be due to this high concentration of FlgE.

10.1128/mBio.00988-18.5 FIG S5  Length distribution of the hook formed inside the IMV (IMV_*flhB* WT-*fliHI*+_). (A) Histograms of the hook length distribution at various FliK concentrations (0 µM, 1 µM, 2 µM, 4 µM, or 8 µM FliK). The concentrations of FlgD, FlgE, the FliH_2_/FliI complex, FliJ, and ATP in the reaction mixture were 4 µM, 4 µM, 1.5 µM, 0.25 µM, and 5 mM, respectively. The concentration of K^+^ (KCl and ATP/2K-KOH) in the reaction mixture was adjusted to 133 mM. The hook length was measured by ImageJ software. (B) An example of a negative-staining electron micrograph of the basal bodies purified from the IMVs (IMV_*flhB* WT-*fliHI*+_) after transport assay. Download FIG S5, TIF file, 2.7 MB. Copyright © 2018 Terashima et al. 2018 Terashima et al. This content is distributed under the terms of the Creative Commons Attribution 4.0 International license.

### ATP hydrolysis by FliI can drive the protein export without PMF.

**FIG 5:**
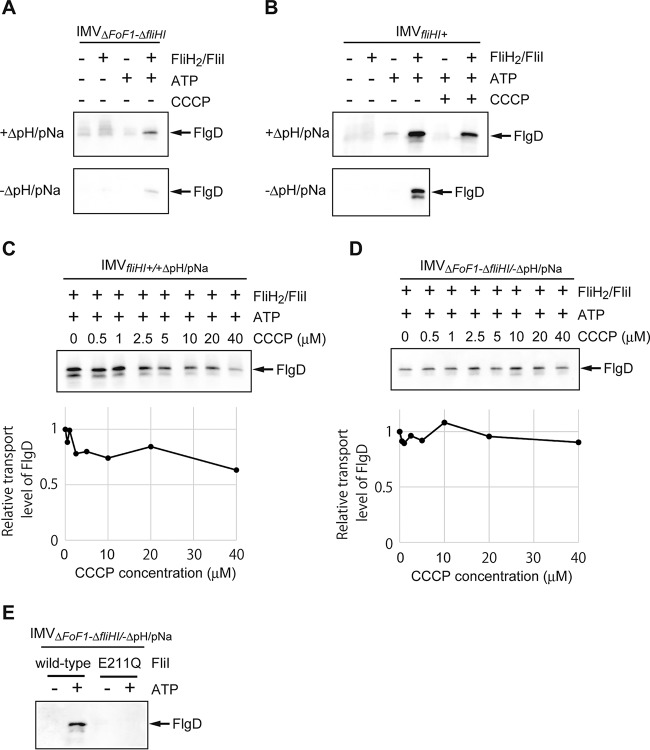
Protein transport driven by ATP hydrolysis. FlgD transported into the IMV was analyzed by immunoblotting. (A) ATP drives the protein transport without PMF. FlgD transport by the IMV_ΔFoF1*-*Δ*fliHI*_ (ΔFoF1*-*Δ*fliHI/*+ΔpH/pNa) (upper panel) and IMV_ΔFoF1*-*Δ*fliHI/−*ΔpH/pNa_ (ΔFoF1*-*Δ*fliHI/−*ΔpH/pNa) (lower panel). The transport assay was conducted with (+) or without (−) the FliH_2_/FliI complex (1.5 µM) and ATP (5 mM). The concentrations of FlgD and FliJ in the reaction mixture were 4 µM and 0.25 µM, respectively. The faint smear bands of the first, second, and third lanes in the upper panel are nonspecific signals. (B) FlgD transport by the normal IMV (*fliHI*^+^*/*+ΔpH/pNa) (upper panel) and IMV_*fliHI*+/−ΔpH/pNa_ (*fliHI*^+^*/−*ΔpH/pNa) (lower panel). The transport assay was conducted with (+) or without (−) the FliH_2_/FliI complex (1.5 µM), ATP (5 mM), and CCCP (10 µM). The concentrations of FlgD and FliJ in the reaction mixture were 4 µM and 0.25 µM, respectively. The FlgD transport of the IMV_*fliHI+*_ was decreased in the presence of CCCP but still remained in the presence of the FliH_2_/FliI complex. The faint smear bands of the first and second lanes in the upper panel are nonspecific signals. (C) The FlgD transport of the normal IMV (IMV_*fliHI+/*+ΔpH/pNa_) in the presence of the FliH_2_/FliI complex and FliJ was suppressed with the increase in the CCCP concentration (upper panel). The transport levels of FlgD relative to that without CCCP are shown (lower panel). The concentrations of FlgD, the FliH_2_/FliI complex, FliJ, and ATP in the reaction mixture were 4 µM, 1.5 µM, 0.25 µM, and 5 mM, respectively. (D) Effect of CCCP on the ATP-driven protein transport (upper panel). The transport levels of FlgD relative to that without CCCP are shown (lower panel). The FlgD transport assay was performed using IMV_ΔFoF1-Δ*fliHI/−*ΔpH/pNa_. The concentrations of FlgD, the FliH_2_/FliI complex, FliJ, and ATP in the reaction mixture were 4 µM, 1.5 µM, 0.25 µM, and 5 mM, respectively. (E) ATP hydrolysis activity of FliI and the protein transport activity in the absence of PMF. The FlgD transport assay was carried out with the FliH_2_/FliI complex (1.5 µM) or the FliH_2_/FliI(E211Q) complex (1.5 µM) with (+) or without (−) ATP (5 mM) using IMV_ΔFoF1*-*Δ*fliHI/−*ΔpH/pNa_. The concentrations of FlgD and FliJ were 4 µM and 0.25 µM, respectively.

As described above, the protein transport by the IMV is mainly driven by PMF generated by the reverse reaction of F_o_F_1_-ATP synthase. However, FlgD transport was still retained to some extent without F_o_F_1_-ATP synthase, even in the absence of the pH and ion concentration gradients, when the FliH_2_/FliI complex, FliJ, and Mg^2+^-ATP were present in the external solution (Fig. 5A), suggesting that protein export can be driven solely by ATP hydrolysis by the FliI ATPase. To confirm protein transport without PMF, we added CCCP in the reaction mixture containing FliJ, the FliH_2_/FliI complex, and Mg^2+^-ATP. The FlgD export activity of the IMV_*fliHI+*_ was reduced in the presence of CCCP but was still significantly retained (Fig. 5B and C). To verify protein transport without PMF, we performed a transport assay using IMV_ΔFoF1-Δ*fliHI*_ without pH and ion concentration gradients (Fig. 5A, lower panel, and D). The FlgD export activity of IMV_ΔFoF1-Δ*fliHI*_ was not affected at all by CCCP up to 40 µM in the presence of FliJ, the FliH_2_/FliI complex, and Mg^2+^-ATP (Fig. 5D). We further tested hook formation without PMF. IMV_ΔFoF1-Δ*fliHI*_ mixed with FlgD, FlgE, FliJ, and the FliH_2_/FliI complex was incubated with or without Mg^2+^-ATP for 1 h. Then, basal bodies were purified from the IMV and observed by negative-staining EM. Polyhooks were formed in the presence of 5 mM ATP and even with 10 µM CCCP but not without ATP (Fig. S6). These results indicate that processive transport of FlgE occurs even in the absence of PMF when FliJ, the FliH_2_/FliI complex, and Mg^2+^-ATP are present. To examine if ATP hydrolysis by FliI is responsible for the protein export without PMF, we added the FliH_2_/FliI(E211Q) complex to the reaction mixture instead of the FliH_2_/FliI complex. FliI(E211Q) can bind Mg^2+^-ATP to form a hexameric ring and associate with the export gate but has no ATP hydrolyzing activity (36). Unlike with wild-type FliI, FlgD was not transported into the IMVs (Fig. 5E). Therefore, we conclude that flagellar protein export can be driven by ATP hydrolysis by FliI in the absence of bulk PMF.

10.1128/mBio.00988-18.6 FIG S6  Negative-staining electron micrographs of the basal bodies purified from the IMVs (IMV_ΔFoF1*-*Δ*fliHI/−*ΔpH/pNa_) after transport assay, without ATP (A), with 5 mM ATP (B), and with 5 mM ATP and 10 µM CCCP (C). No hook structure was formed without ATP (A), but long polyhooks were formed with 5 mM ATP (B), even in the presence of CCCP (C). The concentrations of FlgD, FlgE, the FliH_2_/FliI complex, and FliJ in the reaction mixture were 4 µM, 4 µM, 1.5 µM, and 0.25 µM, respectively. Download FIG S6, TIF file, 2.6 MB. Copyright © 2018 Terashima et al. 2018 Terashima et al. This content is distributed under the terms of the Creative Commons Attribution 4.0 International license.

## DISCUSSION

The flagellar export apparatus transports 20,000 to 30,000 protein subunits of 14 different proteins. Therefore, the coordination of protein export is important to construct the flagellum. The substrate specificity of the export apparatus is switched from rod-hook-type proteins to filament-type proteins after completion of the hook, and the substrate specificity switch is coupled to the gene expression of each type of protein (2). The export of filament-type proteins is coordinated by the flagellar type III export chaperones. These chaperones bind to their specific cognate substrates (37–40). The binding affinity of the chaperone/substrate complexes for FlhA, an export gate protein, is thought to regulate the secretion order of the filament-type proteins (41, 42). Rod-hook-type substrates bind to a well-conserved hydrophobic pocket of the C-terminal cytoplasmic domain of FlhB (43). Recent genetic analyses have suggested that FliH, FliI, FlhA, and FlhB coordinate hook-type protein export with hook assembly to regulate the hook length at 55 nm in *Salmonella* (44). However, the coordination of rod-hook-type protein export was not well known, and no such export chaperones were found for rod-hook-type proteins. We observed that export of FlgE is facilitated by FlgD. FlgD and FlgE are both rod-hook-type export substrates and are supposed to compete for their transport. Thus, FlgE transport should be decreased by an addition of FlgD as seen *in vivo* (45). However, to the contrary, FlgE transport was increased by about two times in the presence of an equal amount of FlgD. This result suggests the presence of a cooperative mechanism between rod-hook-type proteins for efficient and ordered protein export. The enhancement of FlgE export by FlgD is reasonable because the FlgD cap formation is needed prior to the hook formation (29).

The FlgD transport was 20 times enhanced in the presence of 1.5 µM FliH_2_/FliI in the reaction mixture. The number of FliI molecules in a cell was estimated to be 1,500 (46). If we assume that the bacterial cell is a rod-shaped cylinder with a length of 1.5 µm and a diameter of 1 µm capped by hemispherical ends with a diameter of 1 µm, the bacterial cell volume is 1.7 fl, and the concentration of FliI in a cell is estimated to be 1.5 µM. This value is comparable to our experimental condition, implying that a certain amount of the FliH_2_/FliI complex in the cytoplasm is needed for efficient protein export. Then, what is the role of the FliH_2_/FliI complex in solution? *In situ* electron cryotomography revealed that the FliI hexamer complex is associated with the basal-body C-ring through FliH (6, 10, 34), but the FliI hexamer complex has never been observed in the purified basal body *in vitro*, or lysed cells for electron cryotomography (33), indicating that an interaction between the FliH/FliI complex and the basal body is not so strong. Moreover, turnover of FliI between the basal body and the cytoplasmic pool has been demonstrated by fluorescence recovery after photobleaching (FRAP) experiments (34). Thus, a certain amount of the FliH_2_/FliI complex in the cytoplasm may be required for stable association and turnover of the FliI hexamer to the basal body.

Previous *in vivo* studies have revealed that flagellar type III protein export is driven by proton motive force (PMF) and facilitated by ATP hydrolysis by FliI ATPase (18, 19). In agreement with the *in vivo* experiments, protein transport of IMV_Δ*fliHI*_ was observed and inhibited by CCCP. Because the diameter of the central channel of the rod and hook for the substrate passage is only 1.3 nm (47), the substrate proteins must be unfolded during their translocation through the channel. We used purified FlgD and FlgE for a transport assay, and IMV_Δ*fliHI*_ transported these substrate without any help from other proteins except for FliJ (Fig. 2C and 3A). Size exclusion chromatography, which is the final purification step, showed a symmetrical elution peak around the expected molecular weight for each protein. The purified FlgD has been crystallized, and the structure will be published elsewhere. These results indicate that the purified FlgD and FlgE are both well folded in solution. Therefore, our results suggest that the transmembrane gate complex itself has the unfolding activity of substrate proteins. On the other hand, our IMV assay indicates that ATP hydrolysis by FliI can drive the protein export without bulk PMF. Protein transport without PMF was not detected in the previous *in vivo* experiment. In our *in vitro* experiments, the concentration of the substrate was higher than in the *in vivo* experiments and was kept constant. Moreover, the concentrations of the FliH_2_/FliI complex and FliJ were also kept constant. These conditions may enable us to detect the protein transport without PMF. It is still unclear how the cytoplasmic ATPase complex unfolds the export substrate and opens the transmembrane gate. Since the entire structure of the ATPase complex is very similar to that of F_o_F_1_-ATP synthase (8–10), and ATP hydrolysis is closely linked to efficient proton translocation (48), one possibility is that local PMF is generated by the ATPase complex, and the transmembrane gate complex exports the substrates using this local PMF. *In vivo* local pH measurement using pHluorin(M153R) revealed that the ATPase activity of FliI reduced local pH near the export apparatus (48). Moreover, ΔpH is not required for protein export in the presence of FliH and FliI (21). These previous results support this idea, but further investigation is needed to understand the role of ATP hydrolysis by FliI. The Sec protein translocation system requires both PMF and ATP (26), while the TAT system needs only PMF (27). Our study revealed that, unlike these two systems, both PMF and ATP hydrolysis can drive the flagellar type III protein export, although they are both needed for the maximum secretion activity. This energetic redundancy may contribute to the robustness of the flagellar protein export apparatus that is essential for bacterial motility.

Development of an *in vitro* transport assay system with easy control of measurement conditions is important for mechanistic understanding of protein transport. The IMV-based assay has been used for studying various transporters and greatly contributed to the elucidation of the molecular mechanism of substrate transport across the cell membrane (25–27). However, the IMV-based technique has been used only for the transporter consisting of a few to a dozen protein subunits. We applied this technique to the flagellar export machinery, which is composed of more than 250 protein subunits of 12 different types of proteins (FliF, FliG, FliH, FliI, FliJ, FliM, FliN, FliP, FliQ, FliR, FlhA, and FlhB), including the housing of the export apparatus. The export apparatus translocated purified substrate proteins from the external solution into the lumen of the IMV. In addition, the hook length control and the export specificity switching were reproduced in the IMV. These results suggest that the export apparatus in the IMV retains its native function as in the cell. The IMV-based *in vitro* assay described here may also be applied to other large membrane complex systems that are difficult to purify or isolate from the cell membrane in the fully functional form. The *in vitro* system of course cannot fully reproduce the cellular condition, including molecular crowding and complex interactions between various molecules, and therefore the measurement results with the IMV-based system do not directly reflect the cellular events. However, even with such limitations, this IMV-based method will be a useful tool for studying the functional mechanisms and structures of such large complex systems.

## MATERIALS AND METHODS

### Bacterial strains and plasmids.

Bacterial strains and plasmids are listed in Table S1 in the supplemental material. *Salmonella* and Escherichia coli were cultured in LB broth (1% [wt/vol] Bacto tryptone, 0.5% [wt/vol] yeast extract, 0.5% [wt/vol] NaCl). Chloramphenicol was added to a final concentration of 30 µg/ml. Ampicillin was added to a final concentration of 50 µg/ml.

10.1128/mBio.00988-18.7 TABLE S1  Bacterial strains and plasmids used in this study. Download TABLE S1, DOCX file, 0.03 MB. Copyright © 2018 Terashima et al. 2018 Terashima et al. This content is distributed under the terms of the Creative Commons Attribution 4.0 International license.

### Preparation of the cells.

The cells used for IMV preparation were constructed from a *Salmonella* mutant strain with deletion of *flhB*, *flgD*, and *fliT*. The cells were transformed with a pBAD33-based plasmid harboring *flhB*(N269A) and *flhDC*, into the mutant cell. To increase the number of flagellar basal bodies, we deleted *fliT*, the negative regulator of the flagellar class 2 gene, and expressed *flhDC*, the master regulator of the flagellar genes. The *flgD* gene was also deleted, because we selected FlgD as a standard substrate for the transport assay. To prevent the substrate specificity switching from rod-hook-type to filament-type proteins, *flhB* was deleted and the *flhB* N269A mutant gene was introduced. The IMVs used for the hook length control and export switch experiment were prepared from the cells harboring wild-type *flhB* instead of *flhB* N269A.

### Preparation of inverted membrane vesicles.

Overnight cell culture was inoculated into 1 liter of fresh LB broth in a 5-liter flask with 1/100 dilution and cultured at 30°C for 1 h. l-Arabinose was added to the final concentration of 0.02% (wt/vol), and the culture was continued at 18°C for 12 to 16 h (until optical density at 600 nm [OD_600_] reached around 1.5). The cells were collected, suspended into 75 ml of sucrose solution (10 mM Tris-HCl, pH 8.0, 0.75 M sucrose) and stirred with 22.5 mg of lysozyme on ice. Then, 150 ml of 1.5 mM EDTA was poured onto the suspension on ice, and stirring was continued for 1 h at 4°C. The cells were collected at 5,000 × *g* and suspended into 25 ml solution A (20 mM MES-NaOH, pH 6.0, 300 mM NaCl) or solution B (20 mM Tris-HCl, pH 7.5, 125 mM KCl) with 1 tablet of protease inhibitor Complete EDTA-free (Roche). The suspension was passed through a high-pressure cell homogenizer (Stansted) at 90 MPa to produce inverted-membrane vesicles. After removal of debris by centrifugation at 20,000 × *g* for 10 min, IMVs were precipitated by ultracentrifugation at 100,000 × *g* for 1 h. IMVs were suspended into 1 ml of solution A or B and purified by sucrose density gradient centrifugation (60% [wt/wt] 5 ml, 50% [wt/wt] 9 ml, 45% [wt/wt] 9 ml, 40% [wt/wt] 6 ml stepwise gradient in a Beckman ultraclear tube) at 60,000 × *g* (SW32 Ti rotor; Beckman) for 16 h. A brown layer, which is the fraction containing IMVs, was recovered and precipitated by ultracentrifugation at 100,000 × *g* for 1 h. The pellet was suspended into 900 µl of solution A or B, divided into 300-µl aliquots, frozen by liquid nitrogen, and stored at −80°C until use.

### Protein purification.

Cells expressing FlgD, FlgE, or the FliH_2_/FliI complex were suspended in solution C (50 mM Tris-HCl, pH 8.0, 500 mM NaCl) and disrupted by sonication. After removal of the cell debris by centrifugation, the supernatant was loaded onto a HisTrap HP (GE Healthcare) or nickel-nitrilotriacetic acid (Ni-NTA) agarose column (Qiagen), and proteins were eluted with imidazole solution. To remove the His tag, FlgD and FlgE solutions were incubated with thrombin (GE Healthcare) at room temperature for 3 h and then passed through the HiTrap benzamidine FF column (GE Healthcare) to remove thrombin. The FliH_2_/FliI complex was not treated with thrombin; therefore, the His tag of the complex was retained. Finally, proteins were purified using a Superdex 200 column (GE Healthcare) equilibrated with solution B. Cells expressing FliK were suspended in solution D (50 mM Tris-HCl, pH 8.0, 150 mM NaCl) and disrupted by sonication. After removal of the cell debris by centrifugation, supernatant was loaded onto a HisTrap HP (GE Healthcare) column, and proteins were eluted with imidazole solution. To remove the His tag, FliK solution was incubated with thrombin (GE Healthcare) at room temperature for 2 h and then dialyzed with solution A. The FliK solution was passed through the HisTrap HP (GE Healthcare) column to remove His-tag-retaining FliK and then passed through a HiTrap benzamidine FF column (GE Healthcare) to remove thrombin. Finally, proteins were purified using a Superdex 200 column (GE Healthcare) equilibrated with solution B. FliI and FliJ were purified as previously reported in the crystallographic reports (8, 9). Each protein and protein complex eluted as a symmetrical peak corresponding to the expected molecular weight for the products. The purity of the purified proteins was examined by SDS-PAGE.

### Transport assay.

Three hundred microliters of the frozen stock solution of IMVs was thawed at room temperature and homogenized 10 times through an 0.8-µm polycarbonate membrane with the Avanti Mini-Extruder (Avanti Polar Lipids). The IMV solution was loaded on the Sephadex G50 fine column (GE Healthcare) equilibrated with solution B, and the IMV solution was eluted with 1.5 ml of solution B. The transport assay solution was prepared by mixing 100 µl of the IMV solution with 375 µl of external buffer containing Tris-HCl, pH 7.5, KCl, MgCl_2_, dithiothreitol (DTT), and purified substrate proteins, and protein export was initiated by adding 25 µl of 0.1 M ATP solution. The ATP solution was prepared by dissolving ATP (dipotassium salt) in Tris-HCl solution (final concentration of 20 mM) followed by neutralization with KOH. The final reaction mixture contained 20 mM Tris-HCl, pH 7.5, 115 mM or 133 mM KCl, 5 mM MgCl_2_, 1 mM DTT, and 5 mM ATP. The concentration of each chemical component in the external buffer was adjusted to match these values. After incubation at 37°C for 1 h, proteinase K was added at a final concentration of 10 µg/ml to stop the transport reaction, and the reaction mixture was further incubated for 30 min to degrade nontransported substrate molecules. The assay mixture was ultracentrifuged at 100,000 × *g* for 30 min, and the precipitant containing IMV was washed by 1 ml of solution B. After removal of the washing buffer, the precipitant was incubated with 1 ml of solution B containing 10 µg/ml proteinase K for 10 min at room temperature, followed by washing again with 1 ml of solution B, and then IMVs were dissolved with 45 µl of 1% (vol/vol) Triton X-100. The transported proteins were precipitated by trichloroacetate and were detected by immunoblotting analyses with polyclonal anti-FlgD or anti-FlgE antibody.

### Purification of the hook-basal body from IMV.

The hook-basal body complexes were purified according to the method shown by Aizawa et al. with minor modification (49). After the transport reaction, a part of the IMV solution was treated with 0.1% (vol/vol) Triton X-100 and ultracentrifuged (150,000 × *g*, 30 min). The precipitates containing the hook-basal body were suspended in TET solution (10 mM Tris-HCl, pH 8.0, 1 mM EDTA, 0.1% [vol/vol] Triton X-100), and the alkali solution (10% [wt/vol] sucrose, 0.1% [vol/vol] Triton X-100, 0.1 M KCl; pH was adjusted to 11 by KOH) was added to the suspension to completely dissolve the membrane. The suspension was layered on an equal volume of the 35% (wt/vol) sucrose solution prepared by dissolving sucrose in the TET solution in an ultracentrifuge tube, incubated on ice for 30 min, and ultracentrifuged at 38,000 rpm (Beckman TLA100.3 rotor) for 30 min to precipitate the hook-basal body. The precipitates were suspended in the TET solution and then observed by electron microscopy.

### Electron cryotomography.

R0.6/1.0 Quantifoil grids (Quantifoil Micro Tools, Jena, Germany) were glow discharged and pretreated with a solution of 10-nm colloidal gold particles (MP Biomedicals, USA). A 2.6-µl solution of purified IMV was applied to the grid. The grids were blotted briefly with filter paper and then were rapidly plunged into liquid ethane using Vitrobot Mark II (FEI) for freezing. Electron microscopy images were collected at the liquid nitrogen temperature using a Titan Krios electron microscope (FEI) equipped with a field emission gun and a Falcon II direct electron detector (FEI). The microscope was operated at 300 kV and a nominal magnification of ×37,000 with a calibrated pixel size of 4.46 Å after 2 × 2 binning. Images of single-axis tilt series were collected covering an angular range from −70° to +70° with a nonlinear Saxton tilt scheme at 4- to 7-µm underfocus using the Xplore three-dimensional (3D) software package (FEI) and a cumulative dose of ~120 e^−^/Å^2^. The IMOD package (50) was used to align tilted projection images and to generate the final 3D density map from the aligned image stack. The final 3D density map was obtained by the simultaneous iterative reconstruction technique (SIRT).

### Negative-staining electron microscopy.

Sample solutions were applied to carbon-coated copper grids and negatively stained with 2.0% (wt/vol) phosphotungstic acid or 2.0% (wt/vol) uranyl acetate. The samples for the hook length measurement were stained at 4°C to make the hooks straight. Images were observed with a JEM-1011 transmission electron microscope (JEOL, Tokyo, Japan) operating at 100 kV using a TVIPS TemCam-F114 charge-coupled device (CCD) camera or a TemCam-F415 CCD camera.

## References

[1] Macnab RM 2004 Type III flagellar protein export and flagellar assembly. Biochim Biophys Acta 1694:207–217. doi:10.1016/j.bbamcr.2004.04.005. 15546667

[2] Minamino T, Imada K, Namba K 2008 Mechanisms of type III protein export for bacterial flagellar assembly. Mol Biosyst 4:1105–1115. doi:10.1039/b808065h. 18931786

[3] Terashima H, Kawamoto A, Morimoto YV, Imada K, Minamino T 2017 Structural differences in the bacterial flagellar motor among bacterial species. Biophys Physicobiol 14:191–198. doi:10.2142/biophysico.14.0_191. 29362704 PMC5774414

[4] Fukumura T, Makino F, Dietsche T, Kinoshita M, Kato T, Wagner S, Namba K, Imada K, Minamino T 2017 Assembly and stoichiometry of the core structure of the bacterial flagellar type III export gate complex. PLoS Biol 15:e2002281. doi:10.1371/journal.pbio.2002281. 28771466 PMC5542437

[5] Cornelis GR 2006 The type III secretion injectisome. Nat Rev Microbiol 4:811–825. doi:10.1038/nrmicro1526. 17041629

[6] Kawamoto A, Morimoto YV, Miyata T, Minamino T, Hughes KT, Kato T, Namba K 2013 Common and distinct structural features of *Salmonella* injectisome and flagellar basal body. Sci Rep 3:3369. doi:10.1038/srep03369. 24284544 PMC3842551

[7] Fan F, Macnab RM 1996 Enzymatic characterization of FliI: an ATPase involved in flagellar assembly in *Salmonella typhimurium*. J Biol Chem 271:31981–31988. doi:10.1074/jbc.271.50.31981. 8943245

[8] Imada K, Minamino T, Tahara A, Namba K 2007 Structural similarity between the flagellar type III ATPase FliI and F_1_-ATPase subunits. Proc Natl Acad Sci U S A 104:485–490. doi:10.1073/pnas.0608090104. 17202259 PMC1766411

[9] Ibuki T, Imada K, Minamino T, Kato T, Miyata T, Namba K 2011 Common architecture of the flagellar type III protein export apparatus and F- and V-type ATPases. Nat Struct Mol Biol 18:277–282. doi:10.1038/nsmb.1977. 21278755

[10] Imada K, Minamino T, Uchida Y, Kinoshita M, Namba K 2016 Insight into the flagella type III export revealed by the complex structure of the type III ATPase and its regulator. Proc Natl Acad Sci U S A 113:3633–3638. doi:10.1073/pnas.1524025113. 26984495 PMC4822572

[11] Minamino T, Macnab RM 2000 FliH, a soluble component of the type III flagellar export apparatus of *Salmonella*, forms a complex with FliI and inhibits its ATPase activity. Mol Microbiol 37:1494–1503. doi:10.1046/j.1365-2958.2000.02106.x. 10998179

[12] González-Pedrajo B, Fraser GM, Minamino T, Macnab RM 2002 Molecular dissection of Salmonella FliH, a regulator of the ATPase FliI and the type III flagellar protein export pathway. Mol Microbiol 45:967–982. doi:10.1046/j.1365-2958.2002.03047.x. 12180917

[13] Minamino T, Yoshimura SDJ, Morimoto YV, González-Pedrajo B, Kami-ike N, Namba K 2009 Roles of the extreme N-terminal region of FliH for efficient localization of the FliH-FliI complex to the bacterial flagellar type III export apparatus. Mol Microbiol 74:1471–1483. doi:10.1111/j.1365-2958.2009.06946.x. 19889085

[14] Thomas J, Stafford GP, Hughes C 2004 Docking of cytosolic chaperone-substrate complexes at the membrane ATPase during flagellar type III protein export. Proc Natl Acad Sci U S A 101:3945–3950. doi:10.1073/pnas.0307223101. 15001708 PMC374349

[15] Imada K, Minamino T, Kinoshita M, Furukawa Y, Namba K 2010 Structural insight into the regulatory mechanisms of interactions of the flagellar type III chaperone FliT with its binding partners. Proc Natl Acad Sci U S A 107:8812–8817. doi:10.1073/pnas.1001866107. 20421493 PMC2889304

[16] Minamino T, Kinoshita M, Imada K, Namba K 2012 Interaction between FliI ATPase and a flagellar chaperone FliT during bacterial flagellar protein export. Mol Microbiol 83:168–178. doi:10.1111/j.1365-2958.2011.07924.x. 22111876

[17] Minamino T, Kinoshita M, Inoue Y, Morimoto YV, Ihara K, Koya S, Hara N, Nishioka N, Kojima S, Homma M, Namba K 2016 FliH and FliI ensure efficient energy coupling of flagellar type III protein export in *Salmonella*. Microbiologyopen 5:424–435. doi:10.1002/mbo3.340. 26916245 PMC4905995

[18] Minamino T, Namba K 2008 Distinct roles of the FliI ATPase and proton motive force in bacterial flagellar protein export. Nature 451:485–488. doi:10.1038/nature06449. 18216858

[19] Paul K, Erhardt M, Hirano T, Blair DF, Hughes KT 2008 Energy source of flagellar type III secretion. Nature 451:489–492. doi:10.1038/nature06497. 18216859

[20] Minamino T, Morimoto YV, Hara N, Aldridge PD, Namba K 2016 The bacterial flagellar type III export gate complex is a dual fuel engine that can use both H^+^ and Na^+^ for flagellar protein export. PLoS Pathog 12:e1005495. doi:10.1371/journal.ppat.1005495. 26943926 PMC4778876

[21] Minamino T, Morimoto YV, Hara N, Namba K 2011 An energy transduction mechanism used in bacterial flagellar type III protein export. Nat Commun 2:475. doi:10.1038/ncomms1488. 21934659 PMC3195256

[22] Minamino T, Morimoto YV, Kinoshita M, Aldridge PD, Namba K 2014 The bacterial flagellar protein export apparatus processively transports flagellar proteins even with extremely infrequent ATP hydrolysis. Sci Rep 4:7579. doi:10.1038/srep07579. 25531309 PMC4273619

[23] Akeda Y, Galán JE 2005 Chaperone release and unfolding of substrates in type III secretion. Nature 437:911–915. doi:10.1038/nature03992. 16208377

[24] Erhardt M, Mertens ME, Fabiani FD, Hughes KT 2014 ATPase-independent type-III protein secretion in *Salmonella enterica*. PLoS Genet 10:e1004800. doi:10.1371/journal.pgen.1004800. 25393010 PMC4230889

[25] Müller M, Blobel G 1984 *In vitro* translocation of bacterial proteins across the plasma membrane of *Escherichia coli*. Proc Natl Acad Sci U S A 81:7421–7425. doi:10.1073/pnas.81.23.7421. 6390437 PMC392158

[26] Yamane K, Ichihara S, Mizushima S 1987 *In vitro* translocation of protein across *Escherichia coli* membrane vesicles requires both the proton motive force and ATP. J Biol Chem 262:2358–2362. 3029075

[27] Bageshwar UK, Musser SM 2007 Two electrical potential-dependent steps are required for transport by the *Escherichia coli* Tat machinery. J Cell Biol 179:87–99. doi:10.1083/jcb.200702082. 17908913 PMC2064739

[28] Aldridge C, Poonchareon K, Saini S, Ewen T, Soloyva A, Rao CV, Imada K, Minamino T, Aldridge PD 2010 The interaction dynamics of a negative feedback loop regulates flagellar number in *Salmonella enterica* serovar Typhimurium. Mol Microbiol 78:1416–1430. doi:10.1111/j.1365-2958.2010.07415.x. 21143315

[29] Ohnishi K, Ohto Y, Aizawa S, Macnab RM, Iino T 1994 FlgD is a scaffolding protein needed for flagellar hook assembly in *Salmonella typhimurium*. J Bacteriol 176:2272–2281. doi:10.1128/jb.176.8.2272-2281.1994. 8157595 PMC205349

[30] Homma M, Iino T 1985 Locations of hook-associated proteins in flagellar structures of *Salmonella typhimurium*. J Bacteriol 162:183–189. 3884587 10.1128/jb.162.1.183-189.1985 PMC218972

[31] Fraser GM, Hirano T, Ferris HU, Devgan LL, Kihara M, Macnab RM 2003 Substrate specificity of type III flagellar protein export in *Salmonella* is controlled by subdomain interactions in FlhB. Mol Microbiol 48:1043–1057. doi:10.1046/j.1365-2958.2003.03487.x. 12753195

[32] Abrusci P, Vergara-Irigaray M, Johnson S, Beeby MD, Hendrixson DR, Roversi P, Friede ME, Deane JE, Jensen GJ, Tang CM, Lea SM 2013 Architecture of the major component of the type III secretion system export apparatus. Nat Struct Mol Biol 20:99–104. doi:10.1038/nsmb.2452. 23222644 PMC3537844

[33] Chen S, Beeby M, Murphy GE, Leadbetter JR, Hendrixson DR, Briegel A, Li Z, Shi J, Tocheva EI, Müller A, Dobro MJ, Jensen GJ 2011 Structural diversity of bacterial flagellar motors. EMBO J 30:2972–2981. doi:10.1038/emboj.2011.186. 21673657 PMC3160247

[34] Bai F, Morimoto YV, Yoshimura SD, Hara N, Kami-ike N, Namba K, Minamino T 2014 Assembly dynamics and the roles of FliI ATPase of the bacterial flagellar export apparatus. Sci Rep 4:6528. doi:10.1038/srep06528. 25284201 PMC4185386

[35] Moriya N, Minamino T, Hughes KT, Macnab RM, Namba K 2006 The type III flagellar export specificity switch is dependent on FliK ruler and a molecular clock. J Mol Biol 359:466–477. doi:10.1016/j.jmb.2006.03.025. 16630628

[36] Kazetani K, Minamino T, Miyata T, Kato T, Namba K 2009 ATP-induced FliI hexamerization facilitates bacterial flagellar protein export. Biochem Biophys Res Commun 388:323–327. doi:10.1016/j.bbrc.2009.08.004. 19665005

[37] Fraser GM, Bennett JCQ, Hughes C 1999 Substrate-specific binding of hook-associated proteins by FlgN and FliT, putative chaperones for flagellum assembly. Mol Microbiol 32:569–580. doi:10.1046/j.1365-2958.1999.01372.x. 10320579

[38] Auvray F, Thomas J, Fraser GM, Hughes C 2001 Flagellin polymerisation control by a cytosolic export chaperone. J Mol Biol 308:221–229. doi:10.1006/jmbi.2001.4597. 11327763 PMC2528291

[39] Bennett JC, Thomas J, Fraser GM, Hughes C 2001 Substrate complexes and domain organization of the *Salmonella* flagellar export chaperones FlgN and FliT. Mol Microbiol 39:781–791. doi:10.1046/j.1365-2958.2001.02268.x. 11169117 PMC2528293

[40] Aldridge P, Karlinsey JE, Hughes KT 2003 The type III secretion chaperone FlgN regulates flagellar assembly via a negative feedback loop containing its chaperone substrates FlgK and FlgL. Mol Microbiol 49:1333–1345. doi:10.1046/j.1365-2958.2003.03637.x. 12940991

[41] Bange G, Kümmerer N, Engel C, Bozkurt G, Wild K, Sinning I 2010 FlhA provides the adaptor for coordinated delivery of late flagella building blocks to the type III secretion system. Proc Natl Acad Sci U S A 107:11295–11300. doi:10.1073/pnas.1001383107. 20534509 PMC2895114

[42] Kinoshita M, Hara N, Imada K, Namba K, Minamino T 2013 Interactions of bacterial flagellar chaperone-substrate complexes with FlhA contribute to co-ordinating assembly of the flagellar filament. Mol Microbiol 90:1249–1261. doi:10.1111/mmi.12430. 24325251

[43] Evans LD, Poulter S, Terentjev EM, Hughes C, Fraser GM 2013 A chain mechanism for flagellum growth. Nature 504:287–290. doi:10.1038/nature12682. 24213633 PMC3864836

[44] Inoue Y, Morimoto YV, Namba K, Minamino T 2018 Novel insights into the mechanism of well-ordered assembly of bacterial flagellar proteins in *Salmonella*. Sci Rep 8:1787. doi:10.1038/s41598-018-20209-3. 29379125 PMC5789064

[45] Hirano T, Minamino T, Namba K, Macnab RM 2003 Substrate specificity classes and the recognition signal for *Salmonella* type III flagellar export. J Bacteriol 185:2485–2492. doi:10.1128/JB.185.8.2485-2492.2003. 12670972 PMC152621

[46] Dreyfus G, Williams AW, Kawagishi I, Macnab RM 1993 Genetic and biochemical analysis of *Salmonella typhimurium* FliI, a flagellar protein related to the catalytic subunit of the F_o_F_1_ ATPase and to virulence proteins of mammalian and plant pathogens. J Bacteriol 175:3131–3138. doi:10.1128/jb.175.10.3131-3138.1993. 8491729 PMC204635

[47] Fujii T, Kato T, Hiraoka KD, Miyata T, Minamino T, Chevance FFV, Hughes KT, Namba K 2017 Identical folds used for distinct mechanical functions of the bacterial flagellar rod and hook. Nat Commun 8:14276. doi:10.1038/ncomms14276. 28120828 PMC5288503

[48] Morimoto YV, Kamiike N, Miyata T, Kawamoto A, Kato T, Namba K, Minamino T 2016 High-resolution pH imaging of living bacterial cells to detect local pH differences. mBio 7:e01911-16. doi:10.1128/mBio.01911-16. 27923921 PMC5142619

[49] Aizawa SI, Dean GE, Jones CJ, Macnab RM, Yamaguchi S 1985 Purification and characterization of the flagellar hook-basal body complex of *Salmonella typhimurium*. J Bacteriol 161:836–849. 2982790 10.1128/jb.161.3.836-849.1985 PMC214974

[50] Kremer JR, Mastronarde DN, McIntosh JR 1996 Computer visualization of three-dimensional image data using IMOD. J Struct Biol 116:71–76. doi:10.1006/jsbi.1996.0013. 8742726

