# Supplementary figures and images for: In Vitro Reconstitution of Functional Type III Protein Export and Insights into Flagellar Assembly
Source: mBio. 2018 Jun 26;9(3):e00988-18. doi: 10.1128/mBio.00988-18 (PMC6020293; doi:10.1128/mBio.00988-18)

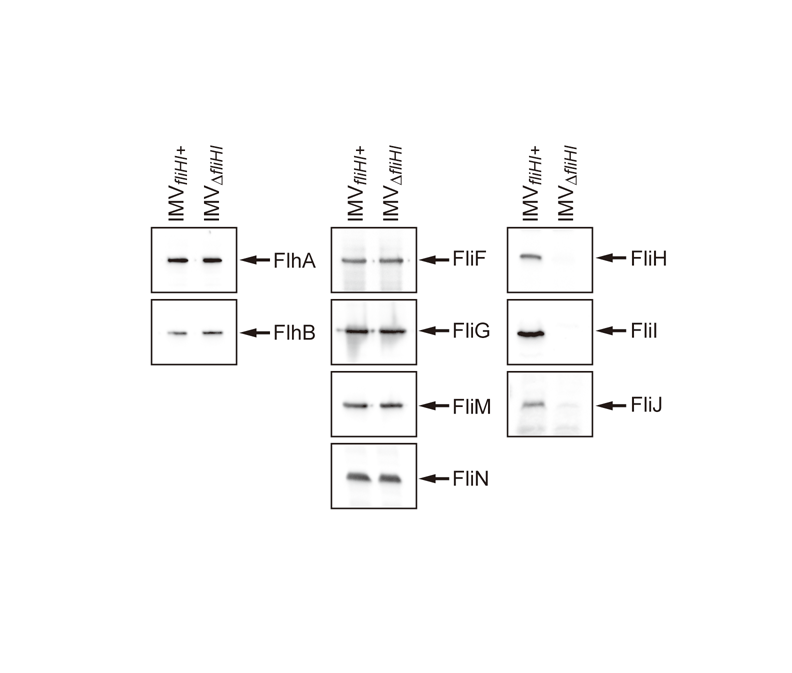

Supplement: FIG S1 [file mbo003183925sf1.tif]

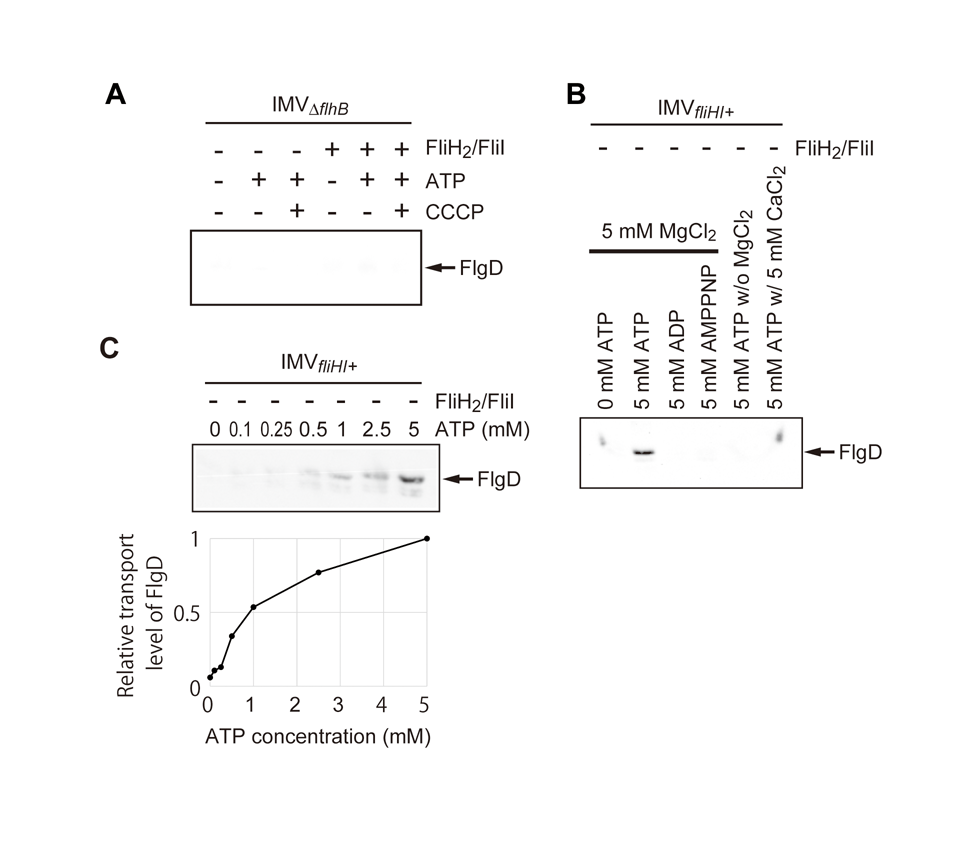

Supplement: FIG S2 [file mbo003183925sf2.tif]

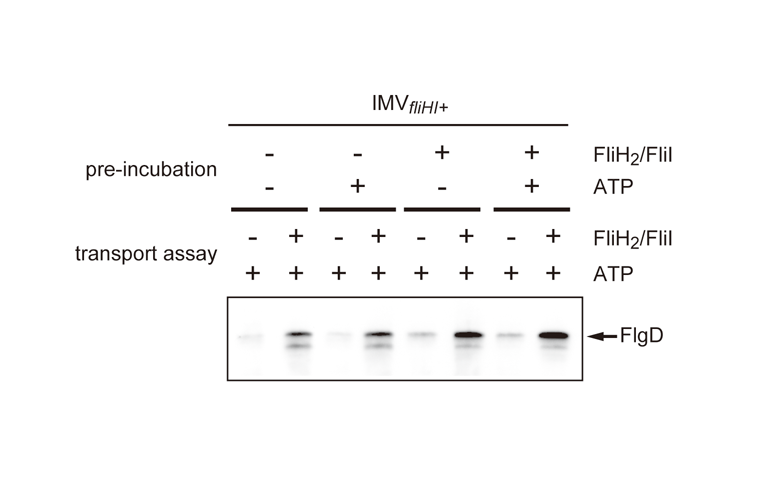

Supplement: FIG S3 [file mbo003183925sf3.tif]

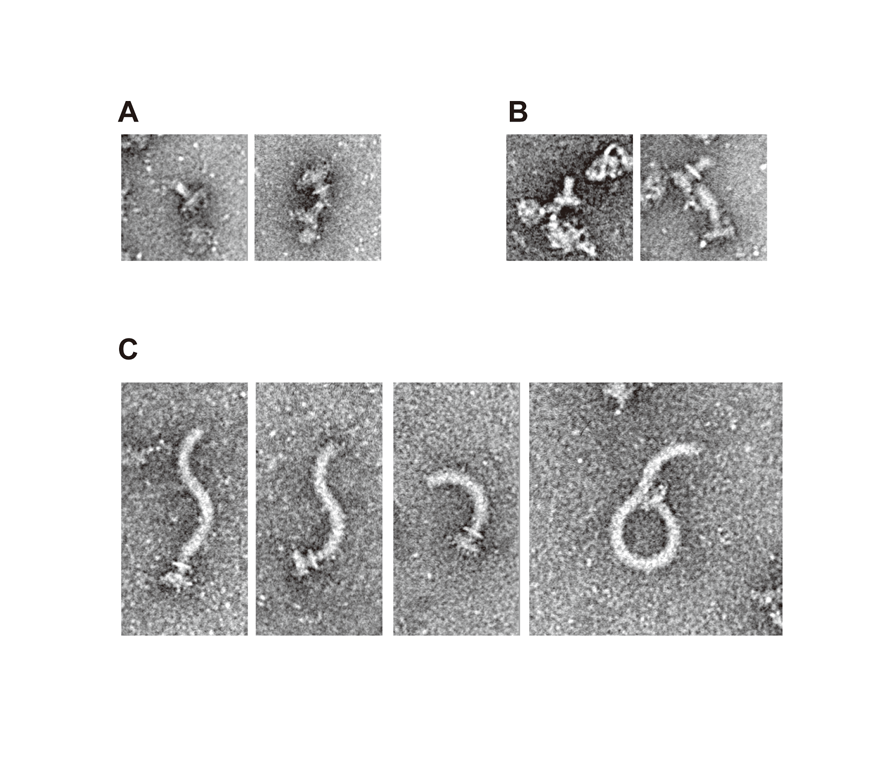

Supplement: FIG S4 [file mbo003183925sf4.tif]

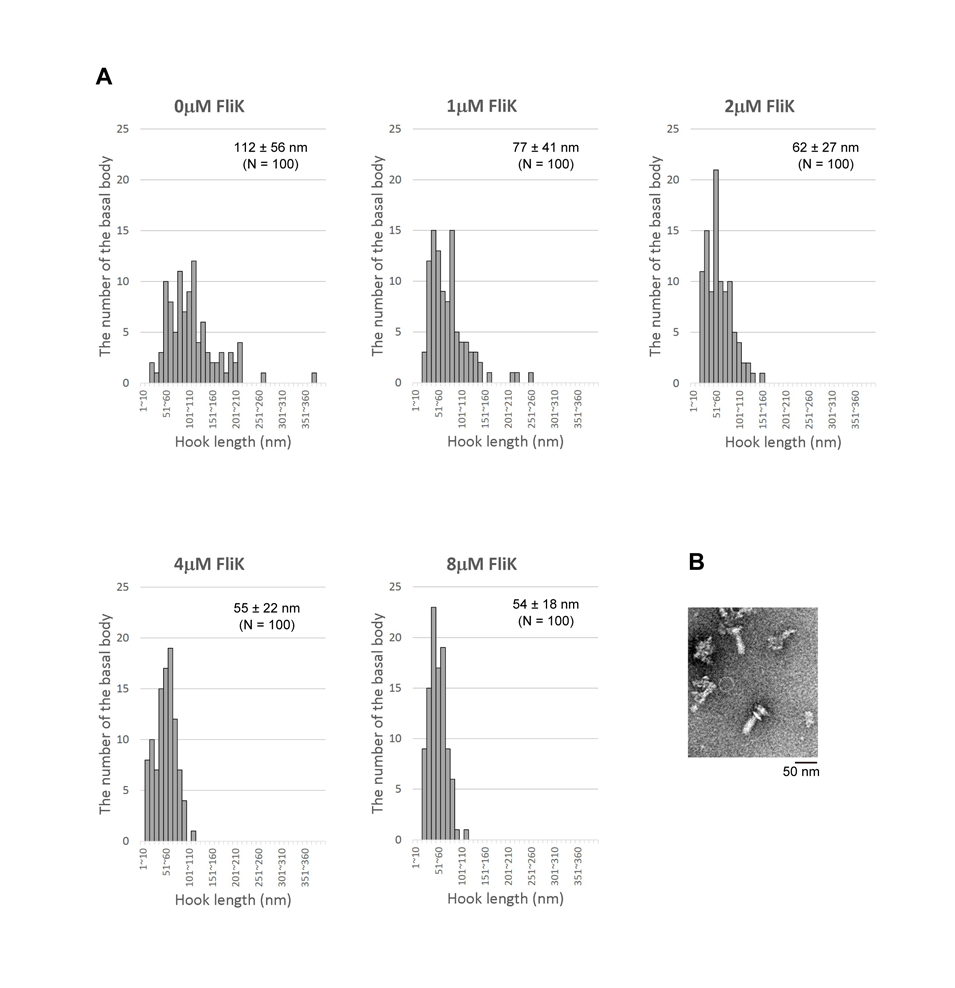

Supplement: FIG S5 [file mbo003183925sf5.tif]

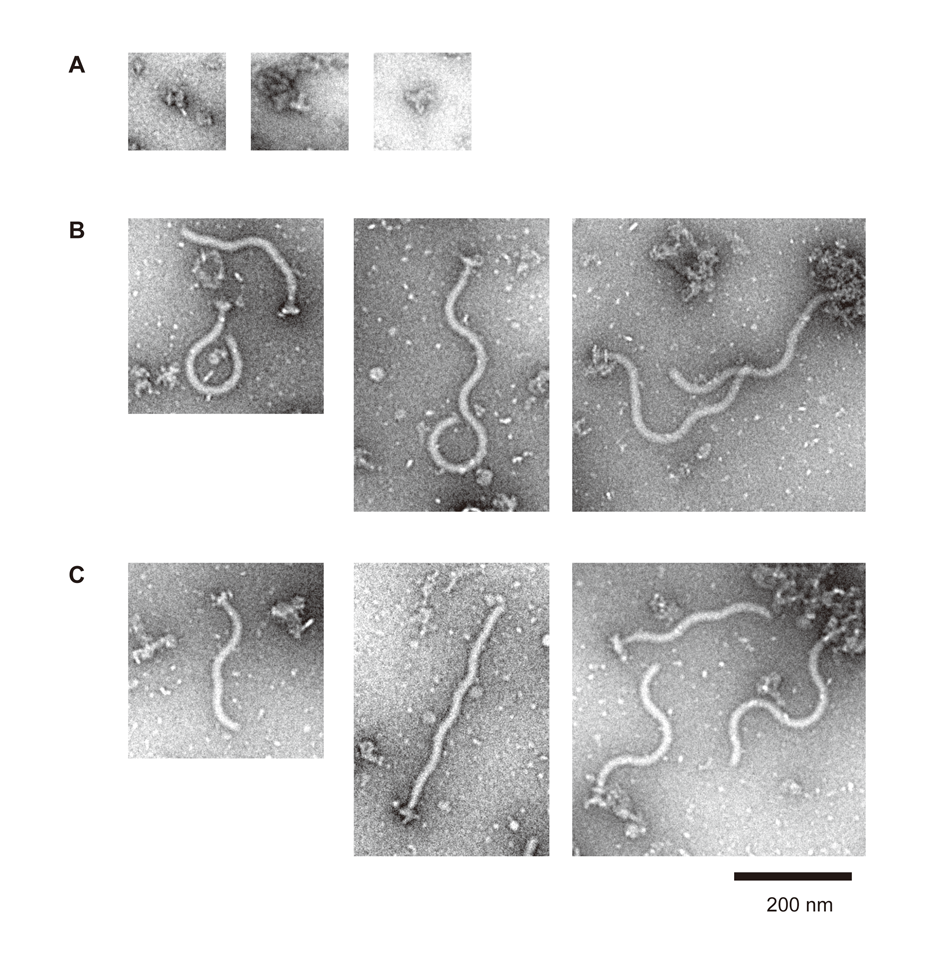

Supplement: FIG S6 [file mbo003183925sf6.tif]
