# Supplementary material for: In Vitro Reconstitution of Functional Type III Protein Export and Insights into Flagellar Assembly
Source: mBio. 2018 Jun 26;9(3):e00988-18. doi: 10.1128/mBio.00988-18 (PMC6020293; doi:10.1128/mBio.00988-18)
Supplement: TABLE S1 [file mbo003183925st1.docx]

**Table S1.** Bacterial strains and plasmids

| Strain or plasmid | Genotype or description | Reference |
| --- | --- | --- |
| *E. coli* strains | | |
| DH5α | F^-^ Φ80d*lacZ* ΔM15 Δ(*lacZYA-argF*)U169 *deoR* *recA*1 *endA*1 *hsdR*17(r_K_^-^, m_K_^+^) *phoA* *supE*44 λ^-^ *thi*-1 *gyrA*96 *relA*1 |  |
| BL21(DE3) | F^-^ *ompT hsd*S_B_ (r_B_^-^ m_B_^-^) *gal dcm* (DE3) | Novagen |
| *Salmonella’s* strains | | |
| MKM50 | ∆*flhB* | (31) |
| STH001 | ∆*flhB* ∆*flgD* ∆*fliT* | In this study |
| STH002 | ∆*flhB* ∆*flgD* ∆*fliT* ∆*liH-fliI* | In this study |
| STH003 | ∆*flhB* ∆*flgD* ∆*fliT* ∆*fliH-fliI* ∆*atpCDGAHFBI*::*tetRA* | In this study |
| Plasmids | | |
| pBAD33 | Cm^r^, expression vector | (51) |
| pBAD33SD | pBAD33-based vector substituted NheI and EcoRI sites (GCTAGCGAATTC) into SD sequence　(GCAGGAGGATTC) | In this study |
| pET3c | Amp^r^, T7 expression vector | Novagen |
| pET15b | Amp^r^, T7 expression vector | Novagen |
| pITH101 | pBAD33SD-*flhB* (FlhB) | This study |
| pITH102 | pET3c-*flhDC* (FlhD/FlhC) | This study |
| pITH103 | pBAD33SD-*flhB* + *flhDC* (FlhB, FlhD/FlhC) | This study |
| pITH104 | pBAD33SD-*flhB*(N269A) + *flhDC* (FlhB(N269A), FlhD/FlhC) | This study |
| pITH105 | pET15b-*flgD* (His-FlgD) | This study |
| pITH106 | pET15b-*flgE* (His-FlgE) | This study |
| pMMIJ001 | pET15b-*fliJ* (His-FliJ) | (9) |
| pMKM1702iH | pTrc99a-*his-fliI* + *fliH* (FliH/His-FliI)*, his-fliI is* derived from pET19b | (10), |
| pMKM1702iH(E211Q) | pTrc99a-*his-fliI(E211Q)* + *fliH* (FliH/His-FliI(E211Q))*, his-fliI is* derived from pET19b | This study |
| pMM1752 | pTrc99a-*his-fliI* (His-FliI)*, his-fliI is* derived from pET15b | This study |
| pITH107 | pET15b-*fliK* (His-FliK) | This study |
| pITH122 | pBAD33SD *flhDC* (FlhD/FlhC) | This study |

51. L. M. Guzman, D. Belin, M. J. Carson, and J. Beckwith, J Bacteriol 177:4121-4130, 1995, doi:10.1128/jb.177.14.4121-4130.1995.
